# Supplementary material for: Thirteen dubious ways to detect conserved structural RNAs
Source: IUBMB Life. Author manuscript; Available in PMC 2024 Jul 10. (PMC11234323; doi:10.1002/iub.2694)
Supplement: supplementary_material [file NIHMS1907110-supplement-supplementary_material.gz › supplemental_material/rscape_v2.0.0.k.tar.gz!/rscape_v2.0.0.k/lib/R2R/R2R-current/NotByZasha/nlopt-2.6.2/src/algs/stogo/paper.pdf]

Global Optimization using Branch-and-Bound

Kaj Madsen and Serguei Zertchaninov[‡]
Department of Mathematical Modelling
Technical University of Denmark

1 Introduction

Many technical and economic problems can be formulated as mathematical programming problems, i.e. as the minimization of a function $f : D \rightarrow \Re$ where $D \subseteq \Re^n$. Very often the function f has several local minima only one of those being interesting, however, namely the smallest one:

$$f^* \equiv inf\{f(x) \mid x \in D\} \tag{1}$$

f^* is called the *global minimum* and the set of points where it is attained is called the set of *global minimizers*.
The problem of constructing an automatic machine (i.e. a computer program) which is guaranteed to find this set has not been solved, and it probably never will be. Interval analytic methods solve the problem for a limited class of problems, as mentioned below. Most other methods either give no guarantee for convergence or convergence is so slow that it in general (i.e. for larger dimensions) is unrealistic. Some techniques are semi-automatic, i.e. it is required that a skilled engineer uses his insight into the technical problem which is modelled by the optimization problem to 'help' the optimization method. Often this can lead to solutions which could not have been found by fully automatic optimization methods. An example of such a technique is given in [1].
In this paper we intend to develop an automatic optimization method. Having the exponential complexity of the global optimization problem in mind

[‡]University of Nizhni Novgorod, Russia

we don't intend to guarantee the solution is found, but rather to try to get the best possible out of a given amount of computational ressources. Thus the user should specify how much computer time and storage he will spend rather than specifying some epsilon accuracy as required by many traditional approaches.

Since the classical deterministic method based on interval arithmetic has been very successful (see e.g. the book of Eldon Hansen, [4]) we base our new technique on the same general branch-and-bound principle, but without the requirement that interval arithmetic must be applicable. Thus the new method is a strategy for managing local methods (like quasi-Newton methods) in a branch-and-bound search for global minimizers.

The basic interval strategy is the following: At any stage of the procedure we assume to have a finite number of

subsets $D_i \subseteq D$

lower bounds, $LB(D_i)$, on $min\{f(x) \mid x \in D_i\}$

upper bounds, $UB(D_i)$, on $max\{f(x) \mid x \in D_i\}$

with the property that any global minimizer is contained in the union of all D_i.

...

Following the interval arithmetic method we assume throughout the paper that D is a compact right parallelipiped parallel to the coordinate axes (denoted a *box*). This assumption could be weakened but it makes the technique much simpler. Furthermore we assume that f is a smooth function (i.e. twice continuously differentiable).

In Section 2 the general branch-and-bound scheme is given, and two realizations of the scheme are described:

(a) The interval method which has guaranteed convergence to the set of global minimizers. Drawback: It is required that an explicit expression for calculating function values $f(x)$ can be supplied.

(b) A new stochastic strategy intended to estimate the interval method without having the need of being rigorous. This method is generally applicable (i.e. finding $f(x)$ may be considered a black box calculation). Drawback: The guaranteed convergence property may be lost.

In Section 3 the new algorithm, based on (b), is presented. Section **??** describes numerical experiments and comparisons with other methods.

2 The general Branch-and-Bound Scheme

We wish to find the global minimum f^* as well as all points x^* for which $f(x^*) = f^*$. Using the notation

$$f(X) \equiv \{f(x) \mid x \in X\}, \qquad X \subseteq D \tag{2}$$

for the range of f over X the problem can be formulated as follows,

$$\begin{array}{ll} \text{find} & f^* = \inf\{f(D)\} \\ \text{and} & X^* = \{x \in D \mid f(x) = f^*\} \end{array} \tag{3}$$

Futhermore, we use the notation C for a finite set of subsets $D_i, i = 1, ..., p$, of D with the properties

$$\begin{array}{c} D_i \text{ is a box} \\ X^* \subseteq US \equiv \cup_{i=1}^{p} D_i \subseteq D \end{array} \tag{4}$$

US is denoted *the candidate set* and the Branch-and-Bound Scheme aims to reduce US and make it converge to X^*. In our description we shall use the notations $P_i \equiv D_i$ if D_i is a point (a degenerate box) and $B_i \equiv D_i$ otherwise, so $C = \{D_i\} = \{P_i\} \cup \{B_i\}$.
Initially $C = \{D\} = \{B_1\}$ assuming the problem is non-trivial. Now the idea is to consider the "smallest" (in some sense related to the function values) non-trivial element B_i from C and try to reduce it without violating (4) . If no reduction takes place, however, then B_i is subdivided into two or more non-trivial pieces. In any case B_i is removed from C, and either the reduced set or the pieces (denoted *result* below) is added to C. Furthermore, a dynamic threshold value *fbound* is used to reduce C: *fbound* is an upper limit for f^* so if for some value of i $LB(D_i) > fbound$ then D_i can be removed from C. Therefore the global minimum is included as follows

$$min\{LB(D_i) \mid D_i \in C\} \leq f^* \leq fbound \tag{5}$$

Thus the model algorithm is the following

$$\text{model algorithm:} \tag{6}$$

initialize $\rightarrow C, fbound$
while not *stop* **do**
  remove-best$(C) \rightarrow B$
  reduce-or-subdivide$(B) \rightarrow result, fbound$
  $C = C \cup \{result\}$
  for $D_i \in C$ **do if** $LB(D_i) > fbound$ **then** $C = C \setminus D_i$
end

Of course the **for**-statement needs not always search the whole of C. This depends on the data structure used. Futhermore, it only needs to be executed when *fbound* has been changed. *stop* is a function which returns true when all elements of C are points or the inclusion (5) of the minimum is sufficiently narrow. Since any known function value can be used as *fbound* ($f^* \leq f(x)$ for any $x \in D$) *fbound* is reduced every time a better function value is found. This basic algorithm was first published by Stig Skelboe [10] who used it in an interval analytic implementation for minimizing rational functions by bisection of subdomains.

2.1 Interval Version

Interval arithmetic and analysis was introduced during the 1960'ies and the standard introductary textbook was authored by Ramon E. Moore, [6]. In interval arithmetic the basic arithmetic operations $\{+, -, *, /\}$ on real numbers are replaced by corresponding operations on real intervals. Furthermore routines for calculating ranges of the standard functions ($sin(x), cos(x), exp(x)$, etc.) are supplied. Therefore, if a real function value $f(b), b \in \Re^n$ can be calculated by a finite number of these operations and standard functions it is straight forward to insert intervals instead of real numbers in the calculation. This provides an *interval function* value $F(B)$ (where B is a box) with the properties

$$\begin{aligned} f(B) &\subseteq F(B) \\ d(f(B), F(B)) &= O(w(B)) \end{aligned} \quad (7)$$

where $w(B)$ denotes the *width* of B,

$$w(B) = \sum_{i=1}^{n} (\overline{b_i} - \underline{b_i})$$

$$B = \{b \mid \overline{b_i} \leq b_i \leq \underline{b_i}\} \quad (8)$$

and d is the distance between two intervals, i.e. the maximum of the distance between corresponding end points. Thus interval arithmetic provides automatic calculation of lower and upper bounds on function ranges, and if the width of B goes to zero then the overestimate disappears at a linear rate. However, for large values of $w(B)$ the overestimate can be quite severe.
The interval global optimization method was basically developed during the 1970'ies ([10],[7],[2],[3]) but since then many researchers have improved the method. A thorough description including theoretical as well as practical aspects can be found in [4]. The basic principle is that lower and upper bounds of function ranges are deducted from the interval function values, e.g. $LB(B)$ is defined as the lower bound of $F(B)$ where F is an interval extension of f, and furthermore $w(B)$ is forced to tend towards zero, if necessary. The general scheme (6) is realized as follows
initialize: $C = \{D\}, fbound = max\{F(D)\}$

$$\mathbf{reduce - or - subdivide}: \quad (9)$$

if *monotone* **then** *result*:= mon(B)
else **if** *Newton-reduction* **then** *result*:= Newton(B)
else *subdivide(B)*→ B^1, B^2
result:= $\{B^1, B^2\}$

monotone: This is tested using an interval extension F' of the gradient f' of f. (... see report, page 4 ff. ...)
...
Subdivide: B is divided into two non-trivial boxes, for instance by halving the side of largest width.

2.2 Stochastic Version

This section describes a stochastic realization of the branch-and-bound scheme (6) which was presented in [5]. The motivation is the success of the interval realization of (6) which is very efficient when it is applicable and when the problem dimension is relatively small. The purpose is to find a new strategy which is applicable to all smooth problems (i.e. in contrast to the

interval method function and gradient calculations are now considered as black boxes). The prize to be paid is that we loose the safety of the interval method: Lower and upper bounds of function ranges cannot be calculated exactly, they must be estimated. Furthermore, like the interval method, we can can certainly not expect the strategy to find all global optimizers of large problems in a realistic amount of time. This is of course due to the exponential nature of the global optimization problem. In Section 3 we present a practical method to cope with that problem.

We now describe the stochastic realization of (6). The variable *fbound* will always contain the smallest function value which has been seen during the iteration. The realization is the following:
remove-best: The box $B_i \in S$ which has the smallest (known) function value is chosen.
reduce-or-subdivide: The scheme (9) is used. It is realized as follows:
monotone: The gradient is calculated at the points $m(B)$ (the mid point of B) and $m(B) \pm \frac{1}{3} * w(B^{(j)})$, j=1, ... ,n, where $B = B^{(1)} \times ... \times B^{(n)}$ and w is the width and at a user specified number of random points in B. Furthermore we calculate the gradient at the point where the line of mean antigradient for all tested points intersects with the border of B.
If there exists at least one coordinate x_i for which $\partial f / \partial x_i(x)$ has constant sign for all points x in consideration, then we decide that no stationary point exists in B. If this decision is correct then the minimum of f over B is attained at one of the sides of B (x_i constant). If this side is interior in D then B can be discarded from further consideration. Otherwise B it can be reduced to the side in question, i.e. the dimension is reduced by one.
Newton-test: In this test a quasi-Newton iteration is used to locate possible local minima in B.
The iteration is started at a number of points, and the idea is as follows: If all iteration sequences go out of B then it is decided that B has no stationary point, and B is discarded or reduced as described above. On the other hand, if all starting points lead to iteration sequences converging to the same point $x \in B$ then we decide that B has exactly one stationary point, and therefore B is discarded and $P = \{x\}$ is added to *result*. A quasi-Newton iteration is not stopped until it either has converged or the iterate is "well out of" B, which in our implementation means

$$\exists j, 1 \leq j \leq n : \mid z_k^{(j)} - m(B^{(j)}) \mid > 0.55 \star w(B^{(j)}) \tag{10}$$

Subdivide: B is divided into two as in the interval version.
LB(B): We use the available information to estimate a lower bound on the function values over a box B. This is done as follows:
Assume p local minima, $x^{(j)}, j = 1, ..., p$, have been located in B, and let $est = min\{f(x^{(j)}) \mid j = 1, ..., p\}$. Let t be a user specified positive integer. If $p \geq t$ then $LB(B) = min\{f(x^{(j)}) \mid j = 1, ..., p\}$ else $LB(B) = -\infty$.

3 The New Method

This section describes the new method which is based on a version of the stochastic strategy of subsection 2.2. Based on our experiences this version is greatly improved as compared with [5] so the convergence is faster and more safe. Furthermore the safety is increased by incorporating the stochastic strategy in an infinite loop where we keep an outer candidate set which always includes the whole of D. Thus we never eliminate boxes in the reduce-and-subdivide step of (6), but if *result* only covers a subset of B then B is added to a garbadge collector (the outer candidate set) supplied with the information we have found about local minima in B. In the outer iteration the garbadge collector forms the basis for a re-defintion of the inner candidate set C: For each box B in the garbadge collector we use the available information about the local mimizers of B in a device which perhaps splits B into several boxes which are all added to C.
The purpose of the outer loop is to provide the possibility of letting the programme run for as long as the user wants. The programme can be stopped at any time providing the best estimate of the global optimizers that has been found. The longer time used, the better result should be expected. For small dimensions, however, the whole set of global minimizers should normally found in a rather modest amount of computer time.
Thus the structure of the new algorithm is the following:

new algorithm: (11)

initialize $\rightarrow C, fbound, G$
while true **do**
    while $C \neq \emptyset$ **do**
        remove-best$(C) \rightarrow B$

reduce-or-subdivide$(B) \rightarrow result, fbound, garbage$
$C = C \cup \{result\}$
$G = G \cup \{garbage\}$
end
while $G \neq \emptyset$ **do**
remove$(G) \rightarrow B$
split-or-reduce$(B) \rightarrow result$
$C = C \cup \{result\}$
end
end

The first **while**-loop differs from the stochastic strategy at the following points:

reduce-or-subdivide: The scheme (9) is used.
monotone: We skip the monotonicity test because this has proved most efficient in our tests. The reason for this is probably the following: After the first loop each full rank box in C contains at least one local minimum (because of *subdivide* below), and thus f is not likely to be monotone in any of these boxes.
Newton-test: The strategy from subsection 2.2 is used. The local method used in our tests is a version of the dog-leg method, [8], which allows for an easy control of the step lengths.
If all iteration sequences go out of B then B is added to *garbage*. If all starting points lead to iteration sequences converging to the same point $x \in B$ then $\{x\}$ is added to *result* and B is added to *garbage*. If none of the two situations take place then we have found several local minima in B and nothing has been added to C or G.
Subdivide: B must contain several known local minima, and the splitting into two is done so each new box contains (known) local minima.
LB(B): First assume $p > 1$ local minima, $x^{(j)}, j = 1, ..., p$, have been located in B. In this case we estimate the Lipschitz constant corresponding to f as follows,

$$Lip = \max_{j \neq i} \frac{(f(x^{(j)}) - f(x^{(i)}))}{\|x^{(j)} - x^{(i)}\|} \tag{12}$$

We use the available information to estimate a lower bound on the function values over a box B. This is done as follows:

Assume p local minima, $x^{(j)}, j = 1, ..., p$, have been located in B, and let $est = min\{f(x^{(j)}) \mid j = 1, ..., p\}$. Let t be a user specified positive integer. If $p \geq t$ then $LB(B) = min\{f(x^{(j)}) \mid j = 1, ..., p\}$ else $LB(B) = -\infty$.

4 Computational Experiments

4.1 Test functions

4.1.1 Box & Betts exponential quadratic sum

Dimension: $n = 3$
Interval: $X = ([0.9, 1.2], [9, 11.2], [0.9, 1.2])$
Formula:

$$f(x) = \sum_{i=1}^{10} \left[exp(-0.1ix_1) - exp(-0.1ix_2) - (exp(-0.1i) - exp(-i))x_3\right]^2$$

Global minimum: 0
Set of minimizers: $(1, 10, 1)$

4.1.2 Rosenbrock function

Dimension: $n = 2$
Interval: $X = [-10, 10]^n$
Formula:

$$f(x) = 100(x_2 - x_1^2)^2 + (1 - x_1)^2$$

Global minimum: 0
Set of minimizers: $(1, 1)$

4.1.3 Shubert function

Dimension: $n = 2$
Interval: $X = [-10, 10]^n$
Formula:

$$f(x) = -\sum_{i=1}^{n} \sum_{j=1}^{5} j sin((j + 1)x_i + j)$$

Global minimum: -24.062499
Set of minimizers:

$(-6.774576, -6.774576)$	$(-6.774576, -0.491391)$	$(-6.774576, 5.791794)$
$(-0.491391, -6.774576)$	$(-0.491391, -0.491391)$	$(-0.491391, 5.791794)$
$(5.791794, -6.774576)$	$(5.791794, -0.491391)$	$(5.791794, 5.791794)$

4.1.4 Paviani function

Dimension: $n = 10$
Interval: $X = [2.001, 9.999]^n$
Formula:

$$f(x) = \sum_{i=1}^{n} \Big(ln^2(x_i - 2) + ln^2(10 - x_i) \Big) - \left(\prod_{i=1}^{n} x_i \right)^2$$

Global minimum: -45.778470
Set of minimizers: $(9.350266, 9.350266, ..., 9.350266)$

4.1.5 Hansen function

Dimension: $n = 2$
Interval: $X = [-10, 10]^n$
Formula:

$$f(x) = \sum_{i=1}^{5} icos((i-1)x_1 + i) \sum_{j=1}^{5} jcos((j+1)x_2 + j)$$

Global minimum: -176.541793
Set of minimizers:

$(-7.589893, -7.708314)$	$(-7.589893, -1.425128)$	$(-7.589893, 4.858057)$
$(-1.306708, -7.708314)$	$(-1.306708, -1.425128)$	$(-1.306708, 4.858057)$
$(4.976478, -7.708314)$	$(4.976478, -1.425128)$	$(4.976478, 4.858057)$

4.1.6 Levy function

Dimension: $n = 4 - 7$
Interval:
for n=4: $X = [-10, 10]^n$
for n=5-7: $X = [-5, 5]^n$
Formula:

$$f(x) = sin^2(3\pi x_1) + \sum_{i=1}^{n-1} (x_i - 1)^2 (1 + sin^2(3\pi x_{i+1})) + (x_n - 1)(1 + sin^2(2\pi x_n))$$

Global minimum:
for n=4: -21.502356
for n=5-7: -11.504403
Set of minimizers:
for n=4: $(1, 1, 1, -9.752356)$
for n=5-7: $(1, ..., 1, -4.754402)$

4.1.7 McCormick function

Dimension: $n = 2$
Interval: $X = ([-1.5, 4], [-3, 4])$
Formula:

$$f(x) = sin(x_1 + x_2) + (x_1 - x_2)^2 - 1.5x_1 + 2.5x_2 + 1$$

Global minimum: -1.9133
Set of minimizers: $(-0.54719, -1.54719)$

4.1.8 Shekel function

Dimension: $n = 4$
Interval: $X = [0, 10]^n$
Formula:

$$f(x) = -\sum_{i=1}^{m} \frac{1}{(x - A_i)(x - A_i)^T + c_i}$$

where for $m = 1$ to 10:

$A_1 = (4, 4, 4, 4)$	$c_1 = 0.1$	$A_6 = (2, 9, 2, 9)$	$c_6 = 0.6$
$A_2 = (1, 1, 1, 1)$	$c_2 = 0.2$	$A_7 = (5, 5, 3, 3)$	$c_7 = 0.3$
$A_3 = (8, 8, 8, 8)$	$c_3 = 0.2$	$A_8 = (8, 1, 8, 1)$	$c_8 = 0.7$
$A_4 = (6, 6, 6, 6)$	$c_4 = 0.4$	$A_9 = (6, 2, 6, 2)$	$c_9 = 0.5$
$A_5 = (3, 7, 3, 7)$	$c_5 = 0.4$	$A_{10} = (7, 3.6, 7, 3.6)$	$c_{10} = 0.5$

Global minimum: $*$
Set of minimizers: $(*)$

4.1.9 Griewank function

Dimension: $n = 10$
Interval: $X = [-500, 700]^n$
Formula:

$$f(x) = \sum_{i=1}^{n} x_i^2/4000 - \prod_{i=1}^{n} cos\left(\frac{x_i}{\sqrt{i}}\right) + 1$$

Global minimum: 0
Set of minimizers: $(0, 0, ..., 0)$

4.1.10 High-dimensional function

Dimension: $n = 30$
Interval: $X = [-20, 30]^n$
Formula:

$$f(x) = -\frac{cos(x^T x)}{1 + x^T x}$$

Global minimum: -1.0
Set of minimizers: $(0, 0, ..., 0)$

4.2 First and last global minima found

Here we offer a table which contains the best results we obtained for the given number of test functions. The table is to show capability of the method to find the solution in case when parameters were chosen to provide the best performance. Although we do not say that these results are the best possible as we have not tested all the reasonable combinations of parameters.

$Function$	$Dim.$	LE	FS	$Start$	$First(F:dF)$	$Last(F:dF)$
4.1.1	3	n	n	1	13 : 7	13 : 7
4.1.2	2	n	n	1	60 : 52	60 : 52
4.1.3	2	y	y	2	31 : 28	5400 : 4650
4.1.4	10	n	n	1	31 : 11	31 : 11
4.1.5	2	y	n	3	182 : 172	5946 : 5550
4.1.6	4	n	n	9	232 : 232	232 : 232
4.1.6	5	n	y	3	645 : 597	645 : 597
4.1.6	6	n	n	5	321 : 305	321 : 305
4.1.6	7	n	y	15	2680 : 2680	2680 : 2680
4.1.7	2	n	n	1	15 : 11	15 : 11
4.1.8	4	n	n	1	28 : 20	28 : 20
4.1.9	10	n	n	3	337 : 301	337 : 301

Using certain uniformity in choosing parameters, namely setting 2N+1 points to start search sequences, and disabling lower bound estimation and fast splitting, we have the following results, which illustrate the case when the best combination of parameters is unknown to the user.

$Function$	$Dim.$	$Total$	$Found$	$First(F:dF)$	$Last(F:dF)$
4.1.1	3	1	1	69 : 69	69 : 69
4.1.2	2	1	1	247 : 247	247 : 247
4.1.3	2	9	8	62 : 62	5733 : 5733
4.1.4	10	1	1	403 : 403	403 : 403
4.1.5	2	9	7	1740 : 1740	11710 : 11710
4.1.6	4	1	1	232 : 232	232 : 232
4.1.6	5	1	1	11010 : 11010	11010 : 11010
4.1.6	6	1	1	2972 : 2972	2972 : 2972
4.1.6	7	1	1	4474 : 4474	4474 : 4474
4.1.7	2	1	1	41 : 41	41 : 41
4.1.8	4	1	1	214 : 214	214 : 214
4.1.9	10	1	1	3412 : 3412	3412 : 3412

Where:
Function - test function number
Dim. - dimensionality
LE - using lower function value estimation
FS - using fast split

Start - number of points to start search sequences
First (F:dF) - number of function:derivative calls to find first minimum
Last (F:dF) - number of function:derivative calls to find last minimum
Total - total number of global minima
Found - number of found global minima

4.3 Analizing parameters

To illustrate how behaviour of the method depends on the parameters... We use function 4.1.3 as an example.

Start	*Random*	*LE*	*FS*	$First(F : dF)$	$Last(F : dF)$
2	0	*n*	*n*	31 : 28	9175 : 7864
2	0	*n*	*y*	31 : 28	6738 : 5787
2	0	*y*	*n*	31 : 28	6441 : 5547
2	0	*y*	*y*	31 : 28	5400 : 4650
5	5	*n*	*n*	65 : 60	8341 : 7536
5	5	*n*	*y*	65 : 60	16580 : 14930
5	5	*y*	*n*	65 : 60	8341 : 7536
5	5	*y*	*y*	65 : 60	16580 : 14930

For 4-dimensional function 4.1.6 were used 4 starting points...

LE	*FS*	$First(F : dF)$
n	*n*	8254 : 7809
n	*y*	379 : 359
y	*n*	8254 : 7809
y	*y*	379 : 359

For function 4.1.5 (y,n) ...

Start	$First(F : dF)$	$Last(F : dF)$
2	54 : 48	6435 : 5475
3	182 : 172	5946 : 5550

Now we will try to form some general recommendations on choosing parameters. User shouldn't assume that we give an ultimate strategy, but we base

our suggestions on the computational experiments we have carried out.

In case of having an unimodal function to be optimized, it is clear that search sequences, ideally, can lead to no more than one point. Hence using lower bound estimation and fast splitting usually makes no sence. User is free to choose the number of random and starting points, but usually it's not necessary to use random sampling, and just a few (even one) starting points are needed to find the solution.

Let us consider multimodal functions. We subdivide them into two classes, first containing functions with unique global minimum, and second containing the rest. Within each class we consider the number of local minima, and separate functions, having relatively few local minima in the region of search, and the rest.

For the functions with unique global minimum and a few local minima, we recommend to use fast splitting combined with small amount of starting points. Using lower bound estimation is usually unnecessary. For the case with many local minima lower bound estimation is more desirable, but fast splitting is not always the best way. Number of starting points can be increased, and random sampling sometimes leads to significant speed up.

For the functions with many global minima method with fast splitting usually finds the first global minimum faster, but slows down in finding the rest. Lower bound estimation sometimes helps to improve this, though it may lead to loosing some of the solutions. Number of starting points should be relatively bigger than in previous cases, and using random points is a good aproach as well.

One significant disadvantage of this method should be pointed out, that is, if object function has a great flatness in the neibourhoods of solution, usually method is unable to reach the prescribed accuracy.

References

[1] J.W. Bandler, R. Biernacki, S. Chen, R. Hemmers, and K. Madsen (1995) *Electromagnetic Optimization Exploiting Aggressive Space Mapping.* IEEE Trans. Microwave Theory Tech., MTT-43, 2874-2882.

[2] E. Hansen (1978) *A global convergent interval analytic method for computing and bounding real roots* BIT 18, 415-424.

[3] E. Hansen (1978) *Global optimization using interval analysis - the multidimentional case* Numerische Mathematik 34, 247-270.

[4] E. Hansen (1992) *Global Optimization using Interval Analysis.* Marcel Dekker, Inc., New York. 230 pages.

[5] K. Madsen (1996) *Real versus Interval Methods for Global Optimization.* Presentation at the Conference "Celebrating the 60th Birthday of M.J.D. Powell", Cambridge, July 1996.

[6] R.E. Moore (1966) *Interval Analysis.* Prentice-Hall, 142 pages.

[7] R.E. Moore (1966) *A test for existence of solutions to non-linear systems.* SIAM Journal on Numerical Analysis 14, 611-615.

[8] M.J.D. Powell (1970) *A Hybrid Method for Non-Linear Equations.* in "Numerical Methods for Nonlinear Algebraic Equations", P. Rabinowitz, ed. 87-114.

[9] A.G.H. Rinnooy Kan and G.T. Timmer (1989) Global Optimization. In "Handbook in Operations Research Vol 1: Optimization", Nemhauser et.al., eds. 631-659.

[10] S. Skelboe (1974) *Computation of Rational Interval Functions.* BIT 14, 87-95.
